# Ulinastatin is effective in reducing mortality for critically ill patients with sepsis: a causal mediation analysis

**DOI:** 10.1038/s41598-018-32533-9

**Published:** 2018-09-25

**Authors:** Qiancheng Xu, Qian Yan, Shanghua Chen

**Affiliations:** Department of Critical Care Medicine, The Second People’s Hospital of Wuhu City, Wuhu, 241000 China

## Abstract

Ulinastatin has been found to have anti-inflammatory effect for patients with sepsis. However, its clinical effects were conflicting. The study aimed to investigate the cost-effectiveness of ulinastatin and to perform mediation analysis to explore the proportion of the total effects that can be explained by inflammatory responses. This is a retrospective study involving critically ill patients with sepsis from January 2014 to July 2017. A total of 263 patients were included in the study, involving 179 patients in the ulinastatin group and 84 in the control group. Ulinastatin group showed significantly lower 28-day mortality rate than that in the control group (31% vs. 55%; p < 0.001). Both total (46330 [26000,83500] vs. 19870 [8747,41140] RMB; p < 0.01) and drug cost (18210 [9492,31920] vs. 7230 [2675,19270] RMB; p < 0.01) were significantly higher in the ulinastatin group than the control group. In multivariable model, the adjusted odds ratio for ulinastatin was 0.304 (95% CI: 0.152 to 0.592; p = 0.001). The mediation analysis showed that the use of ulinastatin was able to reduce the probability of death by 23.5%. The average causal mediation effect of delta C-reactive protein (CRP) was 8%, accounting for 35% of the total effect.

## Introduction

Sepsis is common in the intensive care unit (ICU) and imposes a great challenge for clinicians^1,2^. In recent years, many efforts have been made to increase the awareness of sepsis, as well as to improve the treatment of sepsis^3–5^. While patients with mild sepsis treated outside ICU usually has a good outcome, those requires ICU admission may have mortality rate as high as 50%^6,7^. Such a high mortality is attributable to the development of multiple organ failures such as renal failure, acute respiratory distress syndrome, coagulation dysfunction and cardiac failure. Uncontrolled inflammatory response is the core to the pathophysiology of severe sepsis, which is also a potential target for its treatment^8^. Ulinastatin is one of such agents that have been shown to be promising in improving clinical outcomes for critically ill patients with sepsis^9,10^. However, clinical studies are conflicting. While some studies showed benefits of ulinastatin^11,12^, others failed to replicate the results^13^. A recent systematic review showed no statistical difference on mortality between ulinastatin and control groups^11^. The review included 2 trials with a total sample size of 172, which was underpowered to detect a beneficial effect of ulinastatin. Furthermore, the cost-effectiveness of ulinastatin has never been investigated. In the present study, we aimed to investigate 1) the effectiveness of ulinastatin in reducing mortality for critically ill patients with sepsis; 2) the cost-effectiveness of ulinastatin; and 3) whether the effects of ulinastatin on mortality was mediated by the reduction in the inflammatory biomarker C-reactive protein (CRP).

## Methods

### Setting and subjects

The study was retrospective in design and was conducted in the ICU of Wuhu No. 2 People’s Hospital from January 2014 to July 2017. All patients fulfilled the criteria of sepsis were screened for potential eligibility. According to the sepsis-3.0 definition, sepsis was defined as life-threatening organ dysfunction caused by a dysregulated host response to infection^14^. In our study, organ dysfunction was represented by an increase in the Sequential Organ Failure Assessment (SOFA) score of 2 points or more. Infection was defined as the presence of one of ICD-9 diagnoses for infection such as pneumonia, sepsis, urinary tract infection and blood stream infection. Exclusion criteria included: (1) age younger than 18 years; (2) contraindications for ulinastatin such as allergy; (3) patients with do-not-resuscitation order; (4) pregnant women; and (5) patients transferred to our hospital at late stage of sepsis. The study was approved by the ethics committee of Wuhu NO. 2 People’s Hospital. Informed consent was waived because the study was retrospective in design. The study was performed in accordance to the Helsinki declaration. Individual patient data were de-identified and stored in an encrypted computer.

### Demographic and clinical variables

Demographic data including age and gender were extracted from the first sheet of medical chart. Laboratory variables including white blood cell (WBC), platelet, IL-6, procalcitonin (PCT), C-reactive protein (CRP), and pro-BNP were obtained on day 1 after ICU admission. If there were more than one measurements, the maximum value was recorded. CRP was also obtained on day 3. Changes in CRP (delta CRP) were calculated as the difference between CRP values on day 1 and 3. There were many other inflammatory biomarkers such as PCT, IL-6 measured in our institution. However, they were not included because they are not measured routinely on a daily basis. Acute physiology and chronic health evaluation (APACHE) II and SOFA scores were calculated based on demographic data, vital signs and laboratory variables^15,16^. APACHE II score was calculated by using the clinical and laboratory variables within 24 hours after ICU admission. The values with the maximum point score were used. Patient type included surgical, medical patients and patients admitted from emergency room.

### Use of ulinastatin

The use of ulinastatin (Guangdong Techpool Bio-pharma Co. Ltd., Guangdong, China) was determined by the attending physician and not all sepsis patients received ulinastatin. Data on the use of ulinastatin were extracted from electronic healthcare records. The typical dosage for the use of ulinastatin was 200,000 U three times a day. Ulinastatin would be discontinued if the general condition of the patient was improved, or if there was suspected allergy. Vasopressors including norepinephrine, epinephrine, dopamine greater than 5 mcg/kg/min were recorded.

### Clinical outcomes

Mortality was defined as the vital status at 28 days after ICU admission. Other outcomes included ICU length of stay, duration of mechanical ventilation for the ulinastatin and control groups. ICU length of stay was defined as the period during ICU stay. Readmission to ICU within 48 hours was not considered as successful discharge from ICU. In such a situation, ICU length of stay was calculated as the sum of the two ICU stays. Similarly, reintubation for mechanical ventilation within 48 hours was considered as weaning failure, and the duration of mechanical ventilation was considered as the sum of the two episodes of mechanical ventilation. There were two components of medical cost: 1) total cost was the overall cost during hospital stay; and 2) drug cost was the cost spent on drugs, excluding procedures, laboratory tests, imaging study and other logistic costs.

### Statistical analysis

Statistical descriptions were employed for the overall population, and then for the ulinastatin and control groups separately. Firstly, continuous data were examined by tests for skewness and kurtosis. Normal data were expressed as mean and standard deviation, and were compared between ulinastatin and control groups using student t test. Non-normal data were expressed as median and interquartile range, and were compared using Mann-White U test^17,18^. Logistic regression model was employed to adjust for confounding factors for the effectiveness of ulinastatin on mortality outcome. These factors, as judged by their statistical significance in univariate analysis and subject-matter knowledge, included APACHE II, SOFA, age, use of vasopressors, WBC, neutrophil percent and patient type^19^. Mediation analysis was performed to investigate the proportion of the effect of ulinastatin that was mediated by the change in CRP^20^. Specifically, a linear regression was fit by regressing delta CRP on the use of ulinastatin, then a logistic regression model was fit by regressing 28-day mortality on the use of ulinastatin and delta CRP^21^. The total effects of ulinastatin on mortality was split into two parts, one was the direct effect and the other was the causal mediation effect via delta CRP. All statistical analyses were performed using R (version 3.4.3). Two-tailed p value less than 0.05 were considered as statistically significant.

## Results

**Figure 1:**
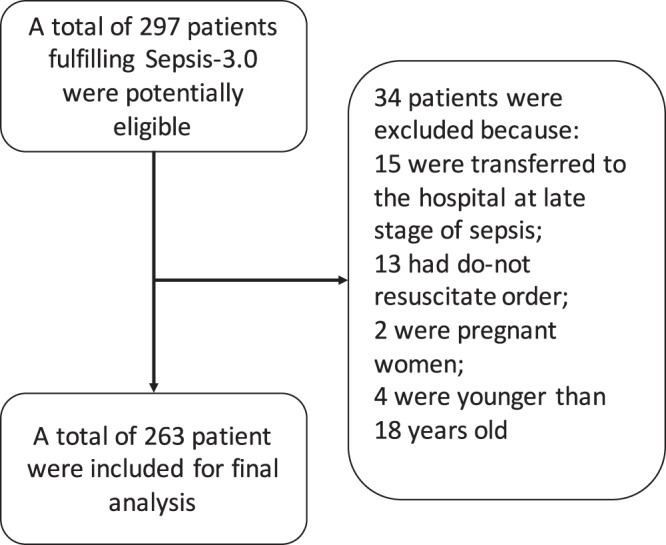
Flowchart of patient selection. Initially, a total of 297 patients who fulfilled the criteria of Sepsis-3.0 were identified by chart review. In-depth screening excluded 34 patients because 13 had do-not-resuscitate order, 15 were transferred from other hospitals to our ICU at late stage of sepsis, 2 were pregnant women and 4 were younger than 18 years old. As a result, a total of 263 patients, including 101 non-survivors and 162 survivors, were included for final analysis.

Initially, a total of 297 patients who fulfilled the criteria of Sepsis-3.0 were identified by chart review. In-depth screening excluded 34 patients because 13 had do-not-resuscitate order, 15 were transferred from other hospitals to our ICU at late stage of sepsis, 2 were pregnant women and 4 were younger than 18 years old (Fig. 1). As a result, a total of 263 patients, including 101 non-survivors and 162 survivors, were included for final analysis. The overall 28-day mortality of 38.4%.

There were 84 patients in the control group and 179 in the ulinastatin group (Table 1). All demographic and clinical variables were not significantly different between the ulinastatin and control groups. Although there was no significant difference in baseline CRP, CRPs measured on day 3 were marginally lower in the ulinastatin group than that in the control group (83.8 [21.35,125.5] vs. 124 [61.9,217]; p = 0.061). However, we found that the patient types were significantly different between the two groups. While there were more medical patients in the control group than that in the ulinastatin group (86% vs. 66%; p = 0.004), there were more surgical patients in the ulinastatin group (19% vs. 8% for elective surgery; 15% vs. 6% for emergency surgery) than the control group.

**Table 1:** Comparisons of baseline characteristics between treatment and control groups.

	Total (n = 263)	Control (n = 84)	Ulinastatin (n = 179)	p
Age (years)	72 (62,80.5)	73 (62,83.25)	72 (62,80)	0.567
Sex (male, Prop.)	175 (0.67)	54 (0.64)	121 (0.68)	0.696
APACHE II	21 (17,26)	19 (16,26)	22 (17,26)	0.624
SOFA	9 (6,12)	9 (5,13)	9 (6,12)	0.719
**Laboratory variables on day 1 (median; IQR)**				
WBC	11.52 (7.25,17.29)	11.74 (7.2,15.92)	11.4 (7.25,18.52)	0.766
Neutrophil percent on day 1 (%)	88.07 (81.34,92.63)	88.41 (81.9,92.7)	87.4 (81.24,92.6)	0.897
Platelet count (*10^9/l)	127 (69,173)	123 (62.25,167.5)	129 (70,173.5)	0.779
Interleukin 6 (pg/ml)	164.3 (46.61,959.4)	130.4 (62.44,947.1)	182.5 (39.83,958.2)	0.822
PCT (ng/ml)	6.39 (1.26,47.09)	8.41 (0.54,37.42)	6.25 (1.54,49.1)	0.726
CRP (mg/l)	133.3 (65.9,215)	138 (68.4,240)	122.5 (53.75,183)	0.198
proBNP (pg/ml)	5142 (1846,15040)	4854 (1468,19740)	5215 (1942,14230)	0.822
CRP on day 3 (mg/l)	104 (49.6,191)	124 (61.9,217)	83.8 (21.35,125.5)	0.061
Vasopressor use	182 (0.69)	60 (0.71)	122 (0.68)	0.695
Type of patients (No. Prop.)				0.004
Elective surgery	32 (0.12)	5 (0.06)	27 (0.15)	
Emergent surgery	41 (0.16)	7 (0.08)	34 (0.19)	
Medical	190 (0.72)	72 (0.86)	118 (0.66)	
Site of infection				0.640
Abdomen	51 (19)	16 (19)	35 (20)	
biliary tract	26 (10)	8 (10)	18 (10)	
lung	101 (38)	31 (37)	70 (39)	
others	10 (4)	1 (1)	9 (5)	
Skin and soft tissue	4 (2)	1 (1)	3 (2)	
Urinary tract	71 (27)	27 (32)	44 (25)	

Abbreviations: APACHE: Acute Physiology and Chronic Health Evaluation; IQR: interquartile; WBC: white blood cell; SOFA: sequential organ failure assessment; PCT: procalcitonin; CRP: C-reactive protein; proBNP: Pro B-Type Natriuretic Peptide; Prop.: proportion.

Note: Chi-square or Fisher’s exact text was employed for comparison of categorical variables. Mann–Whitney U test was used for continuous variables.

Ulinastatin group showed significantly lower 28-day mortality rate than that in the control group (31% vs. 55%; p < 0.001). However, ulinastatin group showed significantly longer duration of mechanical ventilation (3 [1,7] vs. 0 [0, 3] days; p < 0.001), longer length of stay in ICU (5 [3, 11] vs. 1 [0, 6]) and hospital (16 [7, 27] vs. 9.5 [2, 21.25] days; p < 0.001). Both total (46330 [26000,83500] vs. 19870 [8747,41140] RMB; p < 0.01) and drug cost (18210 [9492,31920] vs. 7230 [2675,19270] RMB; p < 0.01) were significantly higher in the ulinastatin group than the control group (Table 2).

**Table 2:** Comparison of outcomes between ulinastatin and control groups.

	Total (n = 263)	Control (n = 84)	Ulinastatin (n = 179)	p
Hospital LOS (days)	14 (6,26)	9.5 (2,21.25)	16 (7,27)	0.004
Duration of MV (days)	2 (0,6)	0 (0,3)	3 (1,7)	0.000
ICU LOS (days)	4 (2,10)	1 (0,6)	5 (3,11)	0.000
Antibiotics duration (days)	11 (5.5,20)	8 (3,19.25)	13 (7,20)	0.003
Duration of vasopressor	2 (0,4)	2 (0,3)	2 (0,4)	0.603
28-day mortality	101 (0.38)	46 (0.55)	55 (0.31)	0.000
Total cost (RMB)	37230 (19870,70690)	19870 (8747,41140)	46330 (26000,83500)	0.000
Drug cost (RMB)	14320 (7051,29160)	7230 (2675,19270)	18210 (9492,31920)	0.000

Note: data were expressed as median and interqurtile range.

Abbreviations: LOS: length of stay; MV: mechanical ventilation; ICU: intensive care unit.

In multivariable logistic regression model (Table 3), the adjusted odds ratio (OR) and confidence interval (CI) for ulinastatin was 0.304 (95% CI: 0.152 to 0.592; p = 0.001). Other variables associated with 28-day mortality were APACHE II (OR: 1.108; 95% CI: 1.030 to 1.199; p = 0.008), age (OR: 1.033; 95% CI: 1.010 to 1.059; p = 0.006) and vasopressor (OR: 4.636; 95% CI: 2.128 to 10.747; p < 0.01).

**Table 3:** Logistic regression model to adjust for confounding factors.

Variables	Odds ratio	Lower limit of 95% CI	Upper limit of 95% CI	p
Age	1.033	1.010	1.059	0.006
APACHE II	1.108	1.030	1.199	0.008
SOFA	1.016	0.899	1.147	0.797
Ulinastatin	0.304	0.152	0.592	0.001
Vasopressor use	4.636	2.128	10.747	0.000
WBC	0.992	0.962	1.015	0.533
Neutrophil percent	1.005	0.988	1.023	0.585
**Patient type (elective surgery as reference)**				
Emergent surgery	0.332	0.074	1.353	0.131
Medical	2.381	0.907	6.762	0.087
**Site of infection (abdomen as reference)**				
Abdomen	0.388	0.105	1.337	0.142
biliary tract	0.738	0.300	1.807	0.505
lung	0.740	0.137	3.782	0.718
others	0.143	0.006	1.747	0.138
Skin and soft tissue	0.980	0.388	2.474	0.965

Abbreviations: APACHE: Acute Physiology and Chronic Health Evaluation; SOFA: sequential organ failure assessment; WBC: white blood cell; CI: confidence interval.

Note: the reported odds ratio corresponds to one unit increase for continuous variables.

**Figure 2:**
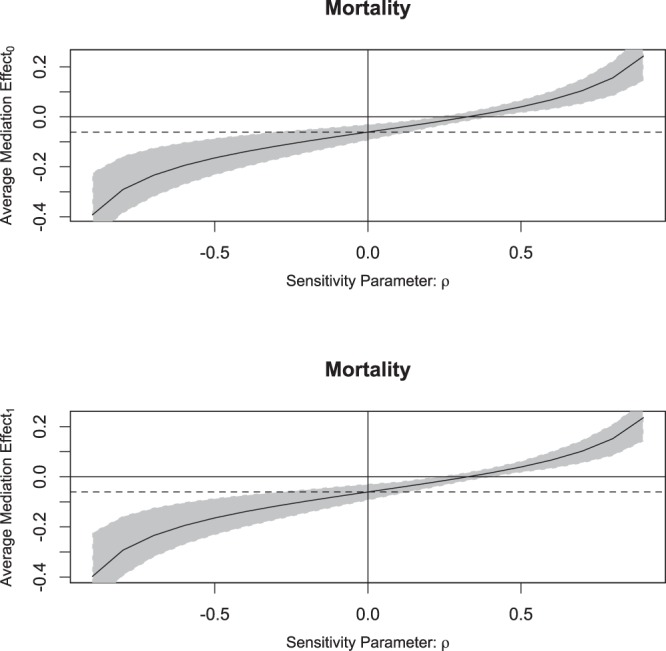
Sensitivity analysis of the causal mediation analysis. Causal mediation analysis is performed under the assumption of sequential ignorability, stating that there was no unmeasured confounder that is related to both the mediator and the outcome. Sensitivity parameter ${\rm{\rho }}$ is the residual correlation and a large value means that there is a large unmeasured confounder. The dashed line indicates the causal mediation effect for the control (upper panel) and treatment groups (lower panel) under assumption that there is no unmeasured confounder (ignorability assumption: ρ = 0). It appears that a ${\rm{\rho }}$ of 0.3 is required to modify the sign of the mediation effect. Judged by subject-matter knowledge, a ${\rm{\rho }}$ value of 0.3 is very large and it is unlikely that the mediation effect doesn’t exist.

The mediation analysis showed that the use of ulinastatin was able to reduce the probability of death by 23.5%. The average causal mediation effect of delta CRP was 8%, accounting for 35% of the total effect. The average direct effect of ulinastatin was 15%, accounting for 65% of the total effect. Since the mediation analysis was performed under the counterfactual framework, the average causal mediation effect and direct effect were also reported for the treated and control groups separately in Table 4. Sensitivity analysis showed that the mediation effect of delta CRP was robust to the violation of ignorability assumption (Fig. 2).

**Table 4:** Mediation analysis with the mediator of C-reactive protein.

	Estimate	95% CI Lower	95% CI Upper	p-value
ACME(control)	−0.0860	−0.1358	−0.0462	0.00
ACME(treated)	−0.0788	−0.1269	−0.0389	0.00
ADE(control)	−0.1560	−0.2602	−0.0340	0.02
ADE(treated)	−0.1489	−0.2500	−0.0322	0.02
Total Effect	−0.2348	−0.3302	−0.1140	0.00
Prop. Mediated(control)	0.3680	0.1674	0.7670	0.00
Prop. Mediated(treated)	0.3385	0.1461	0.7545	0.00
ACME(average)	−0.0824	−0.1320	−0.0420	0.00
ADE(average)	−0.1525	−0.2547	−0.0331	0.02
Prop. Mediated(average)	0.3532	0.1570	0.7607	0.00

Abbreviations: ACME: average causal mediation effect; ADE: average direct effect; Prop.: proportion.

## Discussion

The study showed that the use of ulinastatin was associated with reduced mortality rate in 28-day after ICU admission. The effect remained unchanged after adjustment for potential confounders. However, ulinastatin was associated with increased medical cost. Interestingly, we observed that 35% of the total effect of ulinastatin was mediated via the reduction in CRP, consistent with the anti-inflammatory effect of ulinastatin. The results of the study can be generalized to sepsis population defined by the sepsis-3.0 definition. Furthermore, it was applicable to sepsis patients with any infection site, as the result showed that the infection sites had no significant impact on the effect.

This study was consistent with several previous studies showing that ulinastatin was associated with reduced risk of mortality^22^. In a randomized controlled trial, Karnad and colleagues showed that the 28-day all-cause mortality was 7.3% with ulinastatin (4 deaths) versus 20.3% (12 deaths) with placebo (p = 0.045), and the effect was robust to multivariable adjustment (odds ratio 0.26, 95% CI 0.07–0.95; p = 0.042)^23^. The mortality in Karnad’s study was significantly lower than that in our study, but the beneficial effect of ulinastatin was consistent. This indicates that the beneficial effect of ulinastatin was consistent across a wide range of disease severity. In patients with acute severe pancreatitis, ulinastatin was also found to be able to reduce the mortality rate and the number of new organ dysfunction^24^. However, the beneficial effect was not replicated in elderly patients with multiple organ failure and sepsis^13^. In a randomized controlled trial, Wu and colleagues found that ulinastatin was able to improve inflammatory profile of patients with severe sepsis, but the improvement in mortality outcome was not statistically significant^25^. There are several reasons that are responsible for the discrepancy among these studies. First, the small sample size (n = 60) may explain the failure to obtain a statistically significant result in Wu’s study. The study showed a tendency towards beneficial of ulinastatin on mortality, but the statistical significance was not reached. Second, the selection of study population is important for a study to obtain a positive result. Uchida’s study enrolled elderly patients who had heavy comorbidity burden and poor overall mortality (mean APACHE II = 25). It may be reasonable that ulinastatin alone is not enough to rescue lives of these frail patients. Furthermore, this study enrolled patients with multiple organ failure which was not necessarily caused by sepsis. Third, the evolving definition of sepsis may also explain the conflicting results. While early trials defined sepsis as the infection plus systematic inflammatory response syndrome (SIRS)^23^, more recent trails and the present study enrolled patients with sepsis-3.0 criteria^26^. There is large body of evidence that sepsis identified by these two definitions can be quite different^1,27^.

Medical cost was increased in the ulinastatin group, which can be explained by longer stay in ICU and hospital in this group. Consistent with our study, Abhyankar and colleagues also found the length of stay in ICU and hospital was significantly prolonged for patients receiving ulinastatin^28^. Possibly, patients in the ulinastatin group can survive the critical phase of the disease and the recovery of organ dysfunction required prolonged duration of organ support (e.g. vasopressor use and mechanical ventilation) and stay in ICU. In the control group, patients died in a short period of time. Although the medical cost in the control group was much less than that in the ulinastatin group, the mortality rate was higher. In other studies, although they found significantly reduced mortality rate by the use of ulinastatin, the length of stay in the hospital was not significantly prolonged^22,29^. In a systematic review and meta-analysis, treatment with ulinastatin was not able to reduce the duration of vasopressor use and length of stay in ICU^12^. However, the length of stay in ICU and hospital can be influenced by many factors such as the criteria for ICU discharge, financial insurance of a patient, economic status and culture believes. Thus, direct comparisons between different studies may be problematic due to significant heterogeneity among these trials^11^.

An interesting finding in our study was that the total effect of ulinastatin on mortality outcome can be split into direct and casual mediation effects. The reduction in CRP accounted for 35% of the total effects. CRP was reported in ours study because this biomarker was routinely measured on a daily basis for patients with sepsis. Also, CRP was widely available in other institutions, making it more useful for clinicians. This clinical result further supports results from bench work that ulinastatin has anti-inflammatory effect^30–33^. However, CRP is not a specific biomarker of inflammatory response, and its diagnostic utility is inferior than other inflammatory biomarkers such as interleukins and procalcitonin. Further studies may be needed to investigate whether the remaining 65% direct effect can be explained by these biomarkers, or ulinastatin has other therapeutic effects not involving inflammatory responses. Besides the anti-inflammatory effect of ulinastatin, the direct effect can be explained by its ability to inhibit apoptosis^34^, protect the endothelial glycocalyx layer^35^ and vascular endothelium cells^36^.

Due to retrospective design of the study, some limitations must be acknowledged. First, the selection bias cannot be fully accounted for in observational studies. Although there was no specific indication for the use of ulinastatin in our hospital, the use of ulinastatin cannot be a random process. We have tried to adjust for confounding factors in multivariable regression model, but other unmeasured confounding factors cannot be fully excluded. Second, as mentioned above, CRP is not a specific inflammatory biomarker and it cannot represent the whole process of inflammatory responses. There are hundreds even thousands of inflammatory biomarkers. Thus, the remaining effect of ulinastatin might be explained by these biomarkers. Further studies are needed to address this issue. Third, other agents that have inflammatory modulatory effects were not included for analysis in the present study, making the presence of potential uncontrolled confounding possible. There have been evidence that ulinastain, when combined with other agents such as Xuebijing and thymosin alpha1, can have better effect than used alone^12,37^. These agents were seldom used in our hospital, and thus cannot be analyzed in the present study.

In conclusion, the study found that the use of ulinastatin was associated with reduced 28-day mortality rate in critically ill patients with sepsis. However, ulinastatin was associated with increased length of stay in ICU and hospital, and the medical cost was also increased. Furthermore, we observed that 35% of the total effect of ulinastatin can be explained by the reduction in CRP, consistent with the anti-inflammatory effect of ulinastatin. Further studies are needed to investigate how the remaining 65% direct effect of ulinastatin takes place.

## Declarations

### Ethics approval and consent to participate

The study was approved by the ethics committee of Wuhu NO. 2 People’s Hospital. Informed consent was waived because the study was retrospective in design.
